# Case Report: Heterozygous out-of-frame frameshift variant in *ELANE* without evidence of neutropenia

**DOI:** 10.3389/fimmu.2025.1617868

**Published:** 2025-07-25

**Authors:** Yulin Li, Lang Yu, Yishi Zhang, Liwen Zhang, Lina Zhou, Xuemei Tang, Xiaodong Zhao

**Affiliations:** ^1^ National Clinical Research Center for Child Health and Disorders, Ministry of Education Key Laboratory of Child Development and Disorders, Children’s Hospital of Chongqing Medical University, Chongqing, China; ^2^ Chongqing Key Laboratory of Child Rare Diseases in Infection and Immunity, Children’s Hospital of Chongqing Medical University, Chongqing, China; ^3^ Department of Rheumatology & Immunology, Children’s Hospital of Chongqing Medical University, Chongqing, China

**Keywords:** congenital neutropenia, CYN, SCN, ELANE, frameshift mutation, gene therapy

## Abstract

Mutations in the *ELANE* gene, which encodes neutrophil elastase, are known to cause cyclic neutropenia (CyN) and severe congenital neutropenia (SCN). Currently, targeting *ELANE* for insertion-deletion to trigger nonsense-mediated mRNA decay (NMD) may be a simple and universal method to treat SCN caused by *ELANE* mutations. Here, we present a patient with a heterozygous out-of-frame frameshift variant (-2 frame indel) in exon 4 of the *ELANE* gene. The patient underwent whole exome sequencing during hospitalization for acute parotitis and bronchitis, and found a variant in *ELANE*, c.581_588del AGGCCGGC (p.Q194Rfs*93), inherited from the father. The father of the patient, without neutropenia, did not present any clinically significant symptoms and showed no evidence of somatic mosaicism. During the subsequent three-year follow-up period, the patient did not experience any other major infections, and neutrophil counts remained consistently within the normal range. More importantly, bone marrow cytology indicated mild granulocytic hypoplasia without evidence of maturation arrest. This case provides clinical evidence supporting the notion that late exon frameshift -2 frame indel insertions can inhibit mRNA translation efficiency and prevent the production of mutant proteins, thereby promoting neutrophil maturation. It also bolsters the ongoing development of gene therapy for *ELANE*-related diseases.

## Introduction

The *ELANE* gene codes for neutrophil elastase (NE), a serine protease predominantly localized within neutrophil granules (1). Heterozygous mutations in *ELANE* cause both cyclic (CyN) and severe congenital neutropenia (SCN) (2). While the pathogenic mechanisms of the *ELANE* gene are still being investigated, previous hypotheses suggest that mutant polypeptides encoded by *ELANE* play a significant role. One hypothesis proposes that *ELANE* mutations induce protein misfolding, triggering stress responses and ultimately leading to apoptosis (3, 4). Another hypothesis suggests that mutated peptides are misrouted but still maintain their destructive proteolytic activity (5). Previous observations suggest that the pathogenicity of *ELANE* mutations may be due to gain-of-function alterations in mutant NE, rather than a loss of function in normal NE (5, 6).

Here, we present an intriguing case involving a patient with a heterozygous frameshift variant in exon 4 of the *ELANE* gene. Clinically, the patient does not exhibit symptoms consistent with severe congenital neutropenia or cyclic neutropenia.

## Method

### Genetic diagnosis

Whole-exome sequencing (WES) was performed on genomic DNA of the patient. Mutations in the *ELANE* gene were verified by Sanger sequencing for patient and his parents.

### Construction of plasmids

7.1-pCMV-3×Flag wild-type human *ELANE* plasmids were purchased from Unibio (China) (7). As previously described, Point mutation plasmids and frameshift mutation plasmids were generated by overlap PCR method using WT as template. Mutations were confirmed by Sanger sequencing.

### Western blotting

HEK 293T (ATCC, CRL-11268) were cultured in Dulbecco’s modified Eagle medium (DMEM;Gibco) supplemented with 10% fetal bovine serum (FBS; Excellbio) at 37°C with 5% CO2. Cells were transiently transfected with either wild-type human *ELANE* plasmids or mutant *ELANE* plasmids using PEI (APExBIO) according to the manufacturer’s protocol. The cells were lyzed in radioimmunoprecipitation assay buffer (RIPA) containing phenylmethanesulfonyl fluoride (PMSF) and a protease inhibitor cocktail (PIC). Protein expression was examined by western blotting with an anti-Flag (Sellleck) mouse antibody.

### Detection of mutant *ELANE*-induced ER stress in U2OS cells

U2OS cells (ATCC^®^ HTB-96™) were cultured in DMEM (Gibco) supplemented with 10% fetal bovine serum (FBS; Excellbio) at 37°C with 5% CO_2_. Cells were transiently transfected with either wild-type human *ELANE* plasmids or mutant *ELANE* plasmids using PEI (APExBIO) according to the manufacturer’s protocol. Total RNA was extracted 24 hours post-transfection using TRIzol reagent (Invitrogen). cDNA was synthesized from 1 μg RNA using PrimeScript RT Master Mix (Takara Bio). Quantitative PCR was performed using SYBR Green Supermix (Bio-Rad) with primers specific for ER stress markers (BIP, ATF6) and 18S as an internal control (8).

### Statistical analysis

Samples were compared using two-tailed, unpaired Student’s t test with GraphPad Prism 7.00. Error bars were represented by SD. *P < 0.05, **P < 0.01, ***P < 0.001.

## Results

### Case presentation

**Figure 1:**
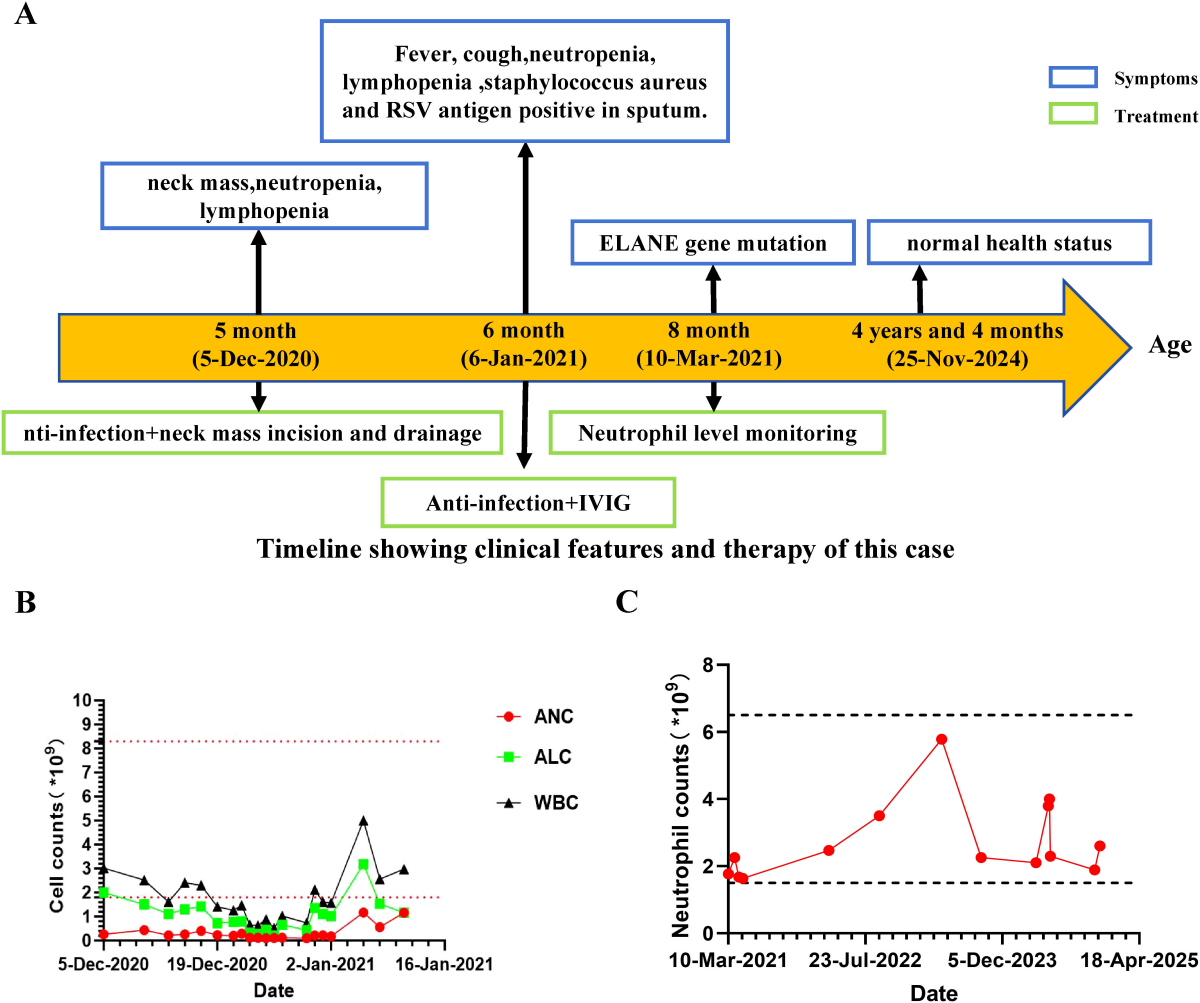
Clinical manifestation of this patient. **(A)** Clinical course of the patient. **(B)** Absolute number of peripheral blood white blood cells (WBC), lymphocytes, and neutrophils during follow-up. Horizontal dashed lines indicate upper and lower reference values of ANC. **(C)** Absolute number of peripheral blood neutrophils during the three-year follow-up.

Here, we present an intriguing case involving a 4-year-and-4-month-old boy born to non-consanguineous parents, exhibiting a heterozygous frameshift variant in exon 4 of the *ELANE* gene, despite lacking evidence of congenital neutropenia (**Figure 1A**). At five months of age, he presented to a local hospital with an left neck mass. Physical examination revealed a left neck mass with mildly elevated local skin temperature, without erythema or ulceration. His hematologic profile revealed a consistent reduction in peripheral blood lymphocytes and neutrophils (Absolute neutrophil count range: 0.16-1.16×10^9^/L, absolute lymphocyte count range: 0.73-3.19×10^9^/L) (**Figure 1B**). Bone marrow cytology suggested mild granulocytic hypoplasia without maturation arrest. Further evaluations, including neck ultrasound and MRI, led to considerations of left acute suppurative parotitis, primary immunodeficiency, and congenital neutropenia. Based on these diagnoses, the local hospital initiated anti-infective treatment and performed incision and drainage of the neck mass. Postoperative purulent puncture culture results were positive for Staphylococcus aureus.

The Patient was admitted to hospital at 6 months of age with a history of intermittent fever for over 1 month, left neck mass for 1 month, and cough for 4 days. Physical examination revealed a body temperature of 36.5°C, hyperpigmentation of the left cervical skin (approximately 6cm × 5cm), and slightly coarse breath sounds bilaterally. Chest CT plain scan demonstrated mild interstitial changes in both lungs. Combined with additional investigations (**Supplementary Figure 1**, **Supplementary Table 1**), the patient was diagnosed with primary immunodeficiency, suppurative parotitis (recovery phase), and acute bronchitis. He has exhibited good growth and development, and during the subsequent three-year follow-up, he has not experienced any major infections apart from occasional upper respiratory tract infections.

### Genetic diagnosis

**Figure 2:**
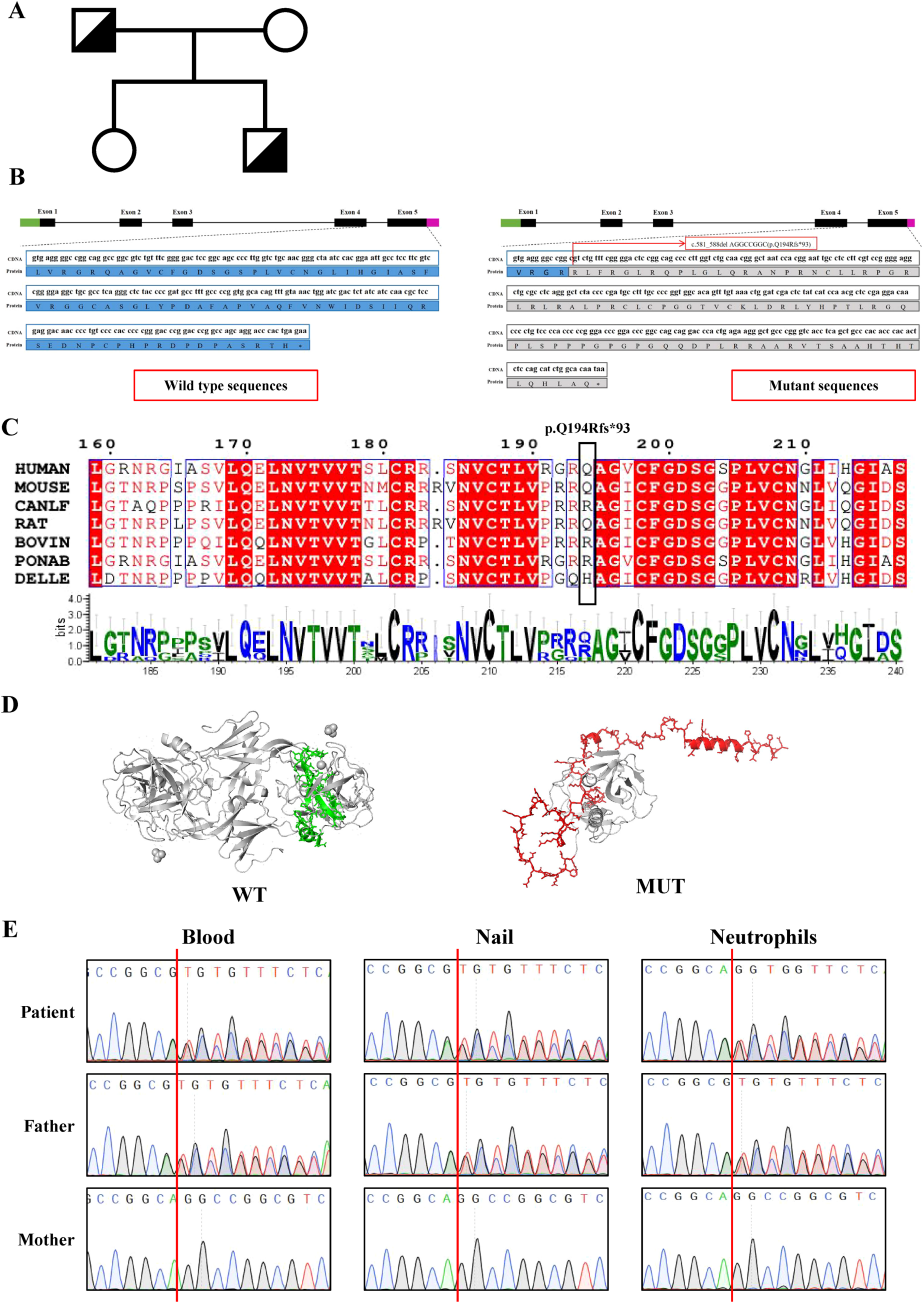
Identification and characterization of *ELANE* mutations in the patient’s family. **(A)** Family pedigree. *ELANE* Q194Rfs*93 mutation in patient and his father is indicated. **(B)** Schematic representation of the *ELANE* gene mutation (c.581_588delAGGCCGGC) and its predicted protein impact. **(C)** Multiple sequence alignment of *ELANE* across seven mammalian species shows complete conservation of the frameshift mutation region (red arrow indicates PQ194Rfs93 site). Conserved catalytic residues are marked with asterisks. **(D)** The wild-type *ELANE* structure (PDB: 7CBK) and AlphaFold3-predicted mutant are shown, with specifically altered residues colored (wild-type: green; mutant: red). **(E)** Sanger sequencing of the *ELANE* gene in patient and family members. Genomic DNA from blood, nails and neutrophils was used.

**Figure 3:**
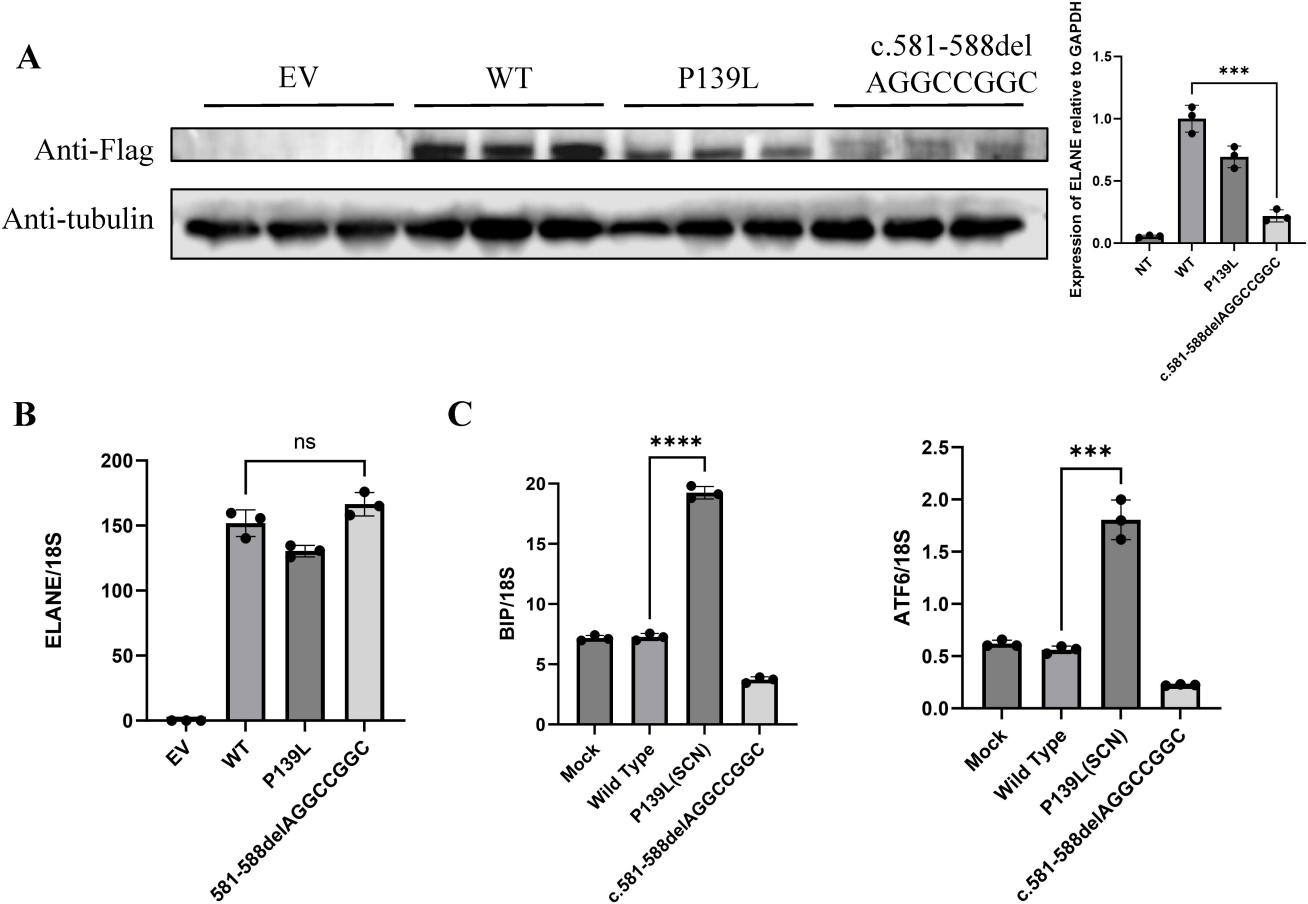
The *ELANE* c.581_588delAGGCCGGC variant lacks pathogenic evidence. **(A, B)** Representative images of mutant *ELANE* protein and mRNA expression across EV, WT, P139L, and c.581-588del AGGCCGGC. from three independent experiments. **(C)** Endoplasmic reticulum stress induction by mutant *ELANE*, representative data from two to three independent experiments are shown. "***" means "p<0.001"; "****" means "p<0.0001", and "ns" means "not significant".

Suspected immune deficiency prompted WES, which identified a novel heterozygous frameshift variant in the *ELANE* gene, c.581_588del AGGCCGGC (p.Q194Rfs*93), inherited from his father (**Figure 2A**, **Supplementary Table 2**). This variant, not previously reported, was predicted to be likely pathogenic (**Figures 2B–D**, **Supplementary Figure 2**). Consistent with ACMG guidelines, this variant is classified as a loss-of-function allele (frameshift mutation) that is expected to disrupt gene function. Its absence in population databases (gnomAD frequency = 0) supports its preliminary classification as Likely Pathogenic (9).This variant lead to an out-of-frame heterozygous frameshift variant in exon 4 of the *ELANE* gene, and the -2 frame indel in the late exon 4 has led to a C-terminal peptide extended by 18 amino acids (aa) (**Figure 2B**, **Supplementary Figure 2**). Given that we failed to obtain sufficient patient samples for analysis, we overexpressed *ELANE* and its mutant variants *in vitro*. The results revealed significantly reduced protein expression in the *ELANE* p.Q194Rfs*93 with normal mRNA levels (**Figures 3A, B**). To validate whether *ELANE* variant-induced protein misfolding triggers endoplasmic reticulum (ER) stress response, we examined the mRNA expression levels of ER stress markers immunoglobulin heavy chain binding protein (BiP) and activating transcription factor 6 (ATF6). Compared with wild-type, human U2OS cells transiently expressing SCN-associated (P139L) *ELANE* mutant exhibited higher expression of both BiP and ATF6, whereas transient expression of this patient’s *ELANE* variant (c.581_588delAGGCCGGC) failed to induce ER stress in U2OS cells (**Figure 3C**).

Notably, the patient’s parents and sister were healthy, with no history of recurrent infections or neutropenia. Particularly, his father had no childhood periodic neutropenia. Mosaic analysis revealed no mosaic phenomenon in the patient or his father (**Figure 2E**).

### Treatment and outcomes

The patient received active anti-infective treatment and intravenous immunoglobulin (IVIG) (supportive treatment during the acute phase of infection), and showed improvement upon discharge. Throughout the course of the illness, a decline in the absolute number of WBC, lymphocytes, and neutrophils was observed in this patient (**Figure 1B**). Interestingly, following the treatment of the patient’s parotitis, the number of peripheral blood lymphocytes and neutrophils gradually increased, and long-term follow-up post-discharge revealed sustained normal absolute neutrophil counts (Absolute neutrophil count range: 1.64-5.78×10^9^/L) (**Figure 1C**). Importantly, he subsequently experienced no significant infection history or need for hematologic disorder treatment.

## Discussions

A notable characteristic of *ELANE* gene mutations is their typically autosomal dominant nature, with the majority of SCN-associated *ELANE* mutations being missense mutations. Despite the presence of a normal *ELANE* allele, the aberrant NE protein still leads to neutropenia (10). Since NE appears non-essential, targeting *ELANE* for indel mutations to trigger NMD may be a straightforward and universal method to treat SCN caused by *ELANE* mutations (11). Previous studies have shown that introducing indels in *ELANE* exon 2 can restore neutrophil maturation in *ELANE*-mutant cells *in vitro (* 12). SCN-associated frameshift mutations are limited to the penultimate exon (exon 4) and the last exon (exon 5) (10). According to the 50-nt rule, these frameshift mutations escape NMD (13). Rao et al. similarly found that efficient gene editing of early exons induced NMD, overcoming the neutrophil maturation arrest in HSPCs of *ELANE*-mutant SCN patients and producing normal hematopoietic engraftment (11). They observed that only -1 frame insertion or deletion (indel) of late exon (exon 4 and exon 5) can lead to the generation of premature termination codons (PTCs), escaping NMD and affecting normal protein function and causing neutrophil maturation arrest. In contrast, a -2 frame indel does not Express mutant protein, allowing normal neutrophil development. Their study demonstrates that a -2 frameshift indel generates additional amino acids, resulting in a shorter 3’ UTR. This alteration causes *ELANE* mRNA to shift from polysomes (actively translating) to monosomes (translationally inactive), leading to reduced translation efficiency. Both *in vivo* and *in vitro* experiments consistently showed significant enrichment of this variant in mature neutrophils.

A review of 26 frameshift mutations in the *ELANE* gene associated with congenital neutropenia revealed that nearly all insertion-deletion mutations lead to a single-frame shift. Moreover, among these 26 mutation types, all except one, which manifested as cyclic neutropenia, were categorized as severe congenital neutropenia (**Table 1**) (10, 14–16). In patient P1, a unique frameshift mutation is observed in the *ELANE* gene. The genotype of P1 is c.269_277del (p.R91Afs*175), occurring in exon 3, resulting in a -2 frame indel. Although P1’s clinical presentation is SCN, P1’s *ELANE* mutation is a compound heterozygous mutation, with the other mutation being a missense mutation in an early exon, c.278A>G (p.E93G) (14). Despite the existence of one patient with a -2 frame indel, owing to the compound heterozygous nature of the mutation and the absence of specific hematologic data, it is still presumed that in previous instances of congenital neutropenia caused by *ELANE* frameshift mutations, the mutations entail single-frame shifts in late exons. Analysis of *ELANE* frameshift variants in gnomAD (**Supplementary Table 3**) reveals distinct positional patterns: early exons harbor diverse frameshift types (0, -1, and -2 frame insertion or deletion), while late exons predominantly exhibit -2 frameshifts. These population frequency observations corroborate the mechanistic framework proposed by Rao et al.

**Table 1:** Frameshift *ELANE* mutations identified in congenital neutropenia patients and Cyclic neutropenia patients.

Frameshifts								
No.	cDNA (NM_001972.2)	Protein (NP_001963.1)	AA length	Indel length	Frame (0, -1 or -2)	Exon	CN	CyN
1	c.269_277del	p.R91Afs∗175	264	-9	-2	3	✓	
2	c.540_541dupCT	p.C181SfsX11	190	+2	-1	4	✓	
3	c.585delC	p.G196AfsX16	210	-1	-1	5	✓	
4	c.583_584dupGC	p.G196PfsX17	211	+2	-1	5	✓	
5	c.588_589dupCG	p.V197AfsX16	211	+2	-1	5	✓	
6	c.589_590insAGGCCGGC	Val197GlufsTer18	213	+8	-1	4		✓
7	c.590_591insTTTTT	p.C198FfsX16	212	+5	-1	5	✓	
8	c.589_596dupGTCTGTTT	p.F199LfsX16	213	+8	-1	5	✓	
9	c.601delG	p.D201TfsX11	210	-1	-1	5	✓	
10	c.601del	p.D201Tfs∗11	210	-1	-1	5	✓	
11	c.602_608delACTCCGG	p.D201AfsX9	208	-7	-1	5	✓	
12	c.601_608dupGACTCCGG	p.S204TfsX11	213	+8	-1	5	✓	
13	c.615delC	p.L206WfsX6	210	-1	-1	5	✓	
14	c.641delG	p.G214EfsX26	238	-1	-1	5	✓	
15	c.649delT	p.S217PfsX23	238	-1	-1	5	✓	
16	c.658delC	p.R220GfsX20	238	-1	-1	5	✓	
17	c.662delG	p.G221EfsX19	238	-1	-1	5	✓	
18	c.667delT	p.C223AfsX17	238	-1	-1	5	✓	
19	c.687delC	p.D230MfsX10	238	-1	-1	5	✓	
20	c.688delG	p.D230MfsX10	238	-1	-1	5	✓	
21	c.689_690insNN	p.D230fsX11	239	+2	-1	5	✓	
22	c.697delG	p.A233PfsX7	238	-1	-1	5	✓	
23	c.697_700delGCCC	p.A233PfsX6	237	-4	-1	5	✓	
24	c.701delC	p.P234RfsX6	238	-1	-1	5	✓	
25	c.703delG	p.V235WfsX5	238	-1	-1	5	✓	
26	c.710delA	p.Q237RfsX3	238	-1	-1	5	✓	

✓ means "yes".

Moreover, patients with *ELANE* gene deletions do not exhibit reduced neutrophil counts, providing additional support for the feasibility of inducing NMD through *ELANE* gene insertions or deletions as a treatment for SCN. Among the 11 documented cases of complete *ELANE* gene deletions, only one patient has been reported to have neutropenia, and this case lacks supporting evidence, suggesting that *ELANE* gene deletions do not lead to decreased neutrophil counts in patients (17). Additionally, a six-month-old Caucasian male patient with a *de novo* nonsense mutation in exon 2 of the *ELANE* gene, predicted to result in protein truncation or NMD, did not present clinically significant neutropenia (18). This absence of neutropenia may be attributed to early exon editing triggering NMD, thereby averting the maturation arrest of neutrophils, consistent with findings by Nasri et al. and Rao (11, 12).

The patient and his father in this case exhibits an out-of-frame heterozygous frameshift variant in exon 4 of the *ELANE* gene, and the -2 frame indel in the late exon 4 has led to a C-terminal peptide extended by 18 amino acids (aa). Our results demonstrated that although mRNA levels remain unchanged, the reduction in protein production reflects inefficient translation, and prevents the generation of mutant proteins, thus supporting neutrophil maturation (**Figures 3A, B**) (11, 13). From another perspective, our failure to observe endoplasmic reticulum stress alterations induced by this mutant variant further substantiates that it does not compromise neutrophil maturation (**Figure 3C**).

It is evident that our patient’s neutropenic episode is associated with severe acute suppurative mumps, as evidenced by the decrease in both white blood cells and lymphocytes. Importantly, these parameters all returned to normal following treatment, and over the three-year follow-up period, no history of periodic neutropenia or other signs of neutropenia were observed in the patient. While we acknowledge the possibility of other potential immune deficiencies in the patient, or that this variant *ELANE* c.581_588del; p.Q194Rfs*93 may reduce the efficiency of immune responses to severe infections (possibilities we cannot entirely exclude at present), we can confirm that both the patient and their father carry an out-of-frame shift variant in the *ELANE* gene (a -2 frame shift corresponding to the end exon), which is not expected to cause congenital neutropenia. These findings support the ongoing development of gene therapy strategies for addressing *ELANE*-associated diseases.

## Data Availability

The datasets generated during and/or analyzed during the current study are available from the corresponding author on reasonable request.
