# Supplementary material for: Case Report: Heterozygous out-of-frame frameshift variant in ELANE without evidence of neutropenia
Source: Front Immunol. 2025 Jul 25;16:1617868. doi: 10.3389/fimmu.2025.1617868 (PMC12331619; doi:10.3389/fimmu.2025.1617868)

**Supplementary materials for “Case Report: Heterozygous Out-of-frame Frameshift Variant in *ELANE* Without Evidence of Neutropenia”**

**Supplemental Methods**

**Respiratory Burst Test**

Respiratory burst was measured by flow cytometry using dihydrorhodamine-1,2,3 (DHR) (Sigma)(1). Peripheral blood samples from the patient and a HC were divided into two reactions of 50 μl each. In one tube, blood was incubated with 18 μl 2 ng/ml PMA (Sigma) and 6 μl 40 mmol DHR, and in the other tube, blood was incubated with 18 μl PBS and 6 μl 40 mmol DHR; both at 37 °C for 20 min. Red blood cells were lysed as described above and cells were re-suspended in 300 μl PBS for analysis.

**Reference**

1. Yu L, Li W, Lv G, Sun G, Yang L, Chen J, Zhou L, Ding Y, Zhang Z, Tang X, et al. De novo somatic mosaicism of CYBB caused by intronic LINE-1 element insertion resulting in chronic granulomatous disease. *J ClinImmunol* (2023) 43:88–100. doi: 10.1007/s10875-022-01347-w

**Supplementary table 1.Other Laboratory features of this patient.**

|  | **Result** | **Reference value** | **Unit** |
| --- | --- | --- | --- |
| **TBNK** |  |  |  |
| CD3+% | 54.63 | 39-73 | % |
| CD3+CD8+% | *32.53* | 11-32 | % |
| CD3+CD4+% | **19.28** | 25-50 | % |
| CD3+CD4+CD8+% | 2.63 | - | % |
| CD3+CD4-CD8-% | 5.45 | - | % |
| NK% | *42.51* | 3-16 | % |
| CD19% | **2.80** | 7-41 | % |
| CD4/CD8 | **0.59** | 0.98-1.94 | - |
| **Other** |  |  |  |
| IgG | 9.1 | 2.86-16.8 | g/L |
| IgA | 0.328 | 0.1-1.29 | g/L |
| IgM | 0.885 | 0.21-1.92 | g/L |
| IgE | 66.3 | 0-165 | IU/ml |
| C3 | 0.9 | 0.79-1.79 | g/L |
| C4 | 0.36 | 0.11-0.61 | g/L |
| CMV-IgG | 168.1 | <6 | AU/ml |
| EBV-NAIgG | Borderline positive | Negative | - |
| EBV-CAIgG | Borderline positive | Negative | - |
| CMV-DNA PCR | positive | Negative | - |

Italics, increased subpopulation; bold, decreased subpopulation

**Supplementary table 2.** Other identified variants were found in WES of this patient.

| **Gene** | **Chromosomal position** | **Reference transcript** | **Nucleic acid change** | **Amino acid chang** | **Het/hom** | **Prediction** |
| --- | --- | --- | --- | --- | --- | --- |
| *TCF3* | chr19-1621929 | NM_0032 00 | c.863C>T | p.P288L | het | Uncertain |
| *C8A* | chr1-57341837 | NM_000562 | c.419G>A | p.C140Y | het | Uncertain |
| *PLCG2* | chr16-81944333 | NM_002661 | c.1934+8T>G | splicing | het | Uncertain |
| *CD19* | chr16-28944321 | NM_001770 | c.445G>A | p.G149R | het | Uncertain |
| *MRTFA* | chr22-40815294 | NM_020831 | c.1448A>G | p.Q483R | het | Uncertain |
| *SLC7A7* | chr14-23243685 | NM_001126106 | c.1123G>A | p.V375M | het | Uncertain |
| *MEFV* | chr16-3304626 | NM_000243 | c.442G>C | p.E148Q | het | Uncertain |

**Supplementary table 3.Frameshift *ELANE* mutations identified in gnomAD and ClinVar.**

|  | | | | | | | | | | | | | | |  |  |  |
| --- | --- | --- | --- | --- | --- | --- | --- | --- | --- | --- | --- | --- | --- | --- | --- | --- | --- |
| ***Frameshifts*** |  | |  | | |  |  | | |  |  |  |  | |  |  |  |
| **No.** | **cDNA (NM_001972.2)** | | | **Protein (NP_001963.1)** | | **AA length** | **Frame**  **(0, -1 or -2)** | | | **Exon** | **ClinVar Germline Classification** | | | | | **Allele Frequency** | |
| 1 | c.16del | | | p.Arg6AspfsTer54 | | 58 | -1 | | | 1 | - | | | | | 6.21e-7 | |
| 2 | c.42dup | | | p.Val15CysfsTer74 | | 87 | -1 | | | 1 | - | | | | | 6.21e-7 | |
| 3 | c.59_60dup | | | p.Leu21CysfsTer40 | | 59 | 0 | | | 1 | - | | | | | 6.21e-7 | |
| 4 | c.91del | | | p.Val31TrpfsTer29 | | 58 | -1 | | | 2 | Likely pathogenic | | | | | 6.26e-7 | |
| 5 | c.92del | | | p.Val31GlyfsTer29 | | 58 | -2 | | | 2 | - | | | | | 6.26e-7 | |
| 6 | c.98dup | | | p.Arg34ProfsTer55 | | 87 | 0 | | | 2 | - | | | | | 6.26e-7 | |
| 7 | c.98del | | | p.Gly33AlafsTer27 | | 58 | -2 | | | 2 | Conflicting classifications of pathogenicity | | | | | 6.26e-7 | |
| 8 | c.131_141del | | | p.Met44ThrfsTer41 | | 83 | -2 | | | 2 | Uncertain significance | | | | | 2.50e-6 | |
| 9 | c.144del | | | p.Gln48HisfsTer12 | | 58 | 0 | | | 2 | - | | | | | 6.27e-7 | |
| 10 | c.205del | | | p.Ala69ArgfsTer7 | | 74 | -1 | | | 2 | Uncertain significance | | | | | 3.82e-6 | |
| 11 | c.236_237dup | | | p.Val80ArgfsTer34 | | 112 | 0 | | | 3 | - | | | | | 6.24e-7 | |
| 12 | c.239_243dup | | | p.Val82CysfsTer33 | | 113 | 0 | | | 3 | - | | | | | 6.24e-7 | |
| 13 | c.239_243del | | | p.Val80GlyfsTer7 | | 85 | -2 | | | 3 | - | | | | | 1.87e-6 | |
| 14 | c.259del | | | p.His87IlefsTer26 | | 111 | -1 | | | 3 | - | | | | | 6.21e-7 | |
| 15 | c.294_295dup | | | p.Phe99CysfsTer15 | | 112 | -1 | | | 3 | - | | | | | 6.21e-7 | |
| 16 | c.321del | | | p.Asn107LysfsTer6 | | 111 | 0 | | | 3 | - | | | | | 6.21e-7 | |
| 17 | c.324del | | | p.Tyr109ThrfsTer4 | | 111 | 0 | | | 3 | - | | | | | 6.20e-7 | |
| 18 | c.333del | | | p.Val112Ter | | 112 | 0 | | | 3 | Uncertain significance | | | | | 6.21e-7 | |
| 19 | c.398del | | | p.Val133GlyfsTer42 | | 173 | -2 | | | 4 | - | | | | | 6.25e-7 | |
| 20 | c.409del | | | p.Gln137SerfsTer38 | | 173 | -1 | | | 4 | - | | | | | 6.24e-7 | |
| 21 | c.437del | | | p.Gly146AlafsTer29 | | 175 | -2 | | | 4 | - | | | | | 6.23e-7 | |
| 22 | c.451_452dup | | | p.Leu152AlafsTer24 | | 174 | -2 | | | 4 | - | | | | | 6.22e-7 | |
| 23 | c.470_492del | | | p.Gly157AspfsTer125 | | 280 | -2 | | | 4 | - | | | | | 2.49e-6 | |
| 24 | c.470del | | | p.Gly157AlafsTer18 | | 173 | -2 | | | 4 | - | | | | | 6.22e-7 | |
| 25 | c.571dup | | | p.Arg191LysfsTer99 | | 288 | -2 | | | 4 | - | | | | | 6.21e-7 | |
| 26 | c.579_589del | | | p.Gln194LeufsTer92 | | 284 | 0 | | | 4 | - | | | | | 6.22e-7 | |
| 27 | c.662dup | | | p.Gly222ArgfsTer68 | | 288 | -2 | | | 5 | - | | | | | 6.20e-7 | |
| 28 | c.772del | | | p.Arg258GlyfsTer? | | - | -1 | | | 5 | - | | | | | 6.20e-7 | |
| 29 | c.799del | | | p.His267ThrfsTer? | | - | -2 | | | 5 | - | | | | | 1.24e-6 | |
| 30 | c.796_797insG | | | p.Thr266SerfsTer24 | | 288 | -2 | | | 5 | - | | | | | 6.20e-7 | |

**Supplementary Figure 1.** DHR-1,2,3 flow cytometry results and variations of patient. Unstimulated (blue line); PMA-stimulated (red line).


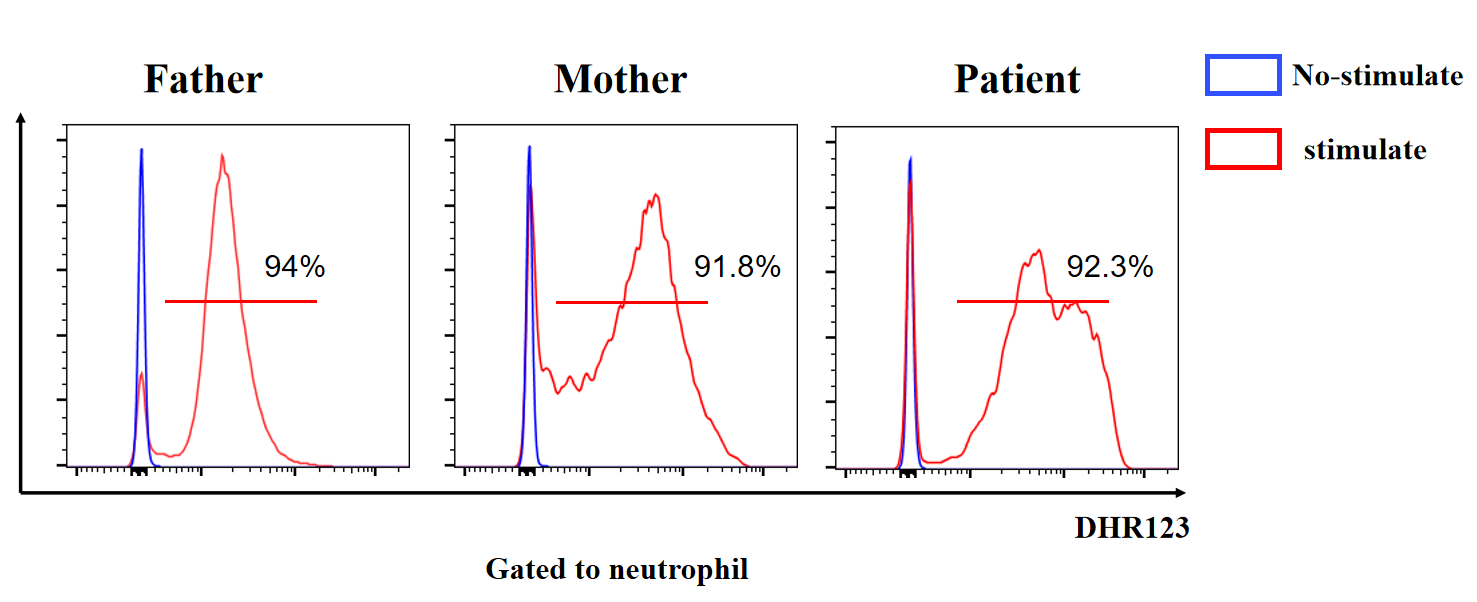


**Supplementary Figure 2.** The mutant protein structure was predicted using AlphaFold3, with ipTM score not available (ipTM=-) and pTM score of 0.63. The color scheme represents different prediction confidence levels,Dark blue: Very high confidence (pLDDT > 90);Light blue: Confident (90 > pLDDT > 70);Yellow: Low confidence (70 > pLDDT > 50);Orange: Very low confidence (50 > pLDDT).


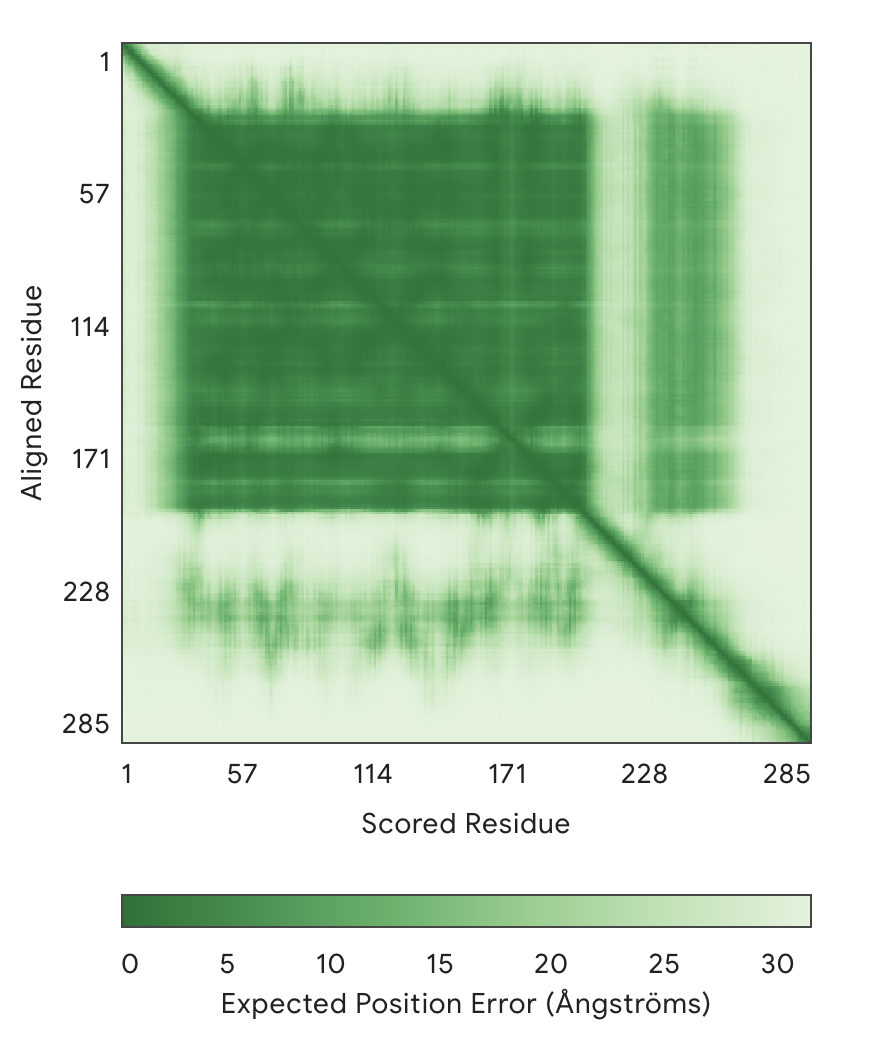

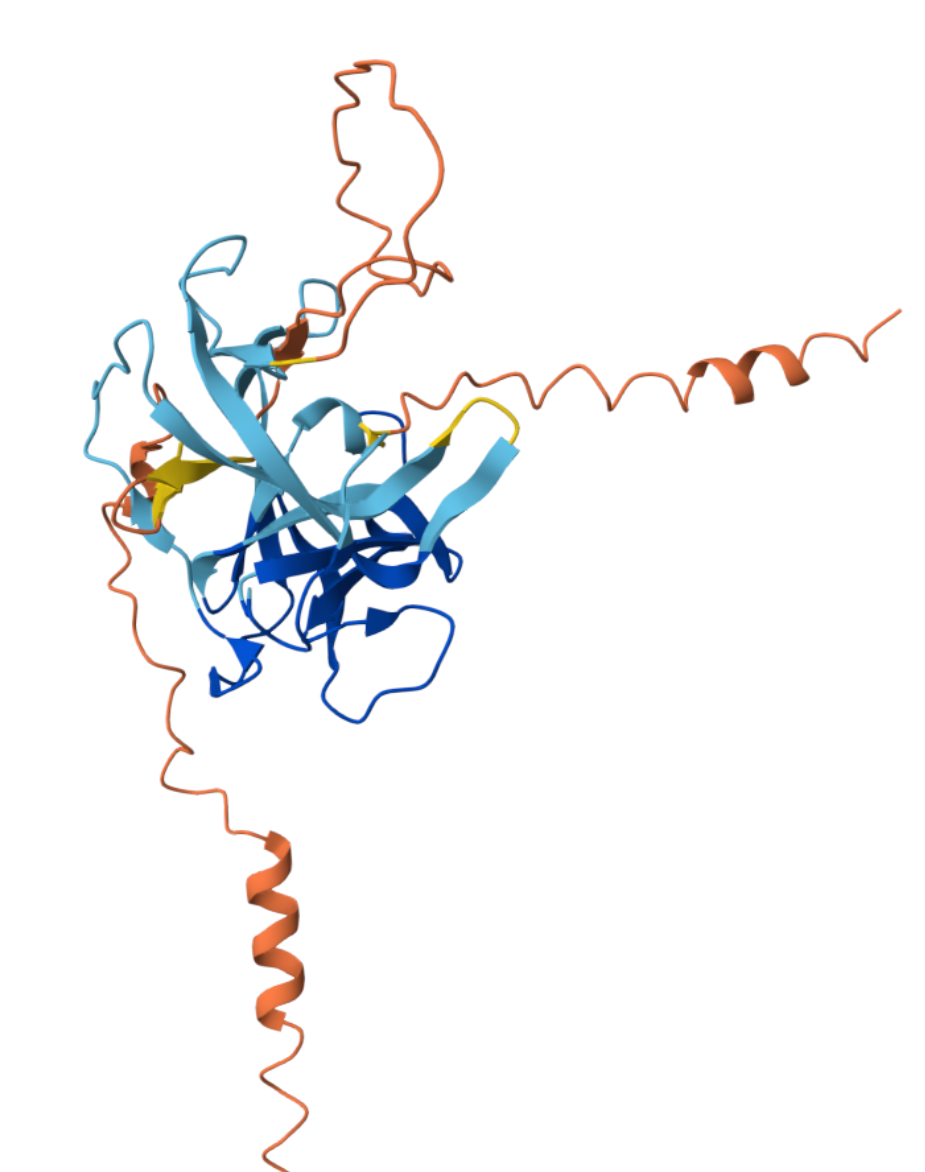

Supplement: Supplementary file 1 [file DataSheet1.docx]
